# Rapid progression of chorioretinal atrophy in punctate inner choroiditis: a case report

**DOI:** 10.1186/s13256-021-03169-7

**Published:** 2021-12-15

**Authors:** Yuka Kasuya, Yuji Inoue, Satoru Inoda, Yusuke Arai, Hidenori Takahashi, Hidetoshi Kawashima, Yasuo Yanagi

**Affiliations:** 1 Department of Ophthalmology, Jichi Medical University, 3311-1 Yakushiji, Shimotsuke-shi, Tochigi, 329-0431 Japan; 2 Department of Ophthalmology, Asahikawa Medical University, 1-1-1 Higashinijou, Midorigaoka, Asahikawa-shi, Hokkaido 078-8510 Japan; 3 Medical Retina, Singapore National Eye Centre, Singapore, Singapore; 4 Medical Retina, Singapore Eye Research Institute, Singapore, Singapore; 5 The Ophthalmology & Visual Sciences Academic Clinical Program, Duke-NUS Medical School, National University of Singapore, Singapore, Singapore

**Keywords:** Punctate inner choroiditis, Chorioretinal atrophy, Rapid progression, Case report

## Abstract

**Background:**

The chorioretinal inflammatory lesions occurring in punctate inner choroiditis evolve into punched-out atrophic scars. Typically, the progression is gradual. We report a case of highly myopic punctate inner choroiditis with rapid progression of chorioretinal atrophy.

**Case presentation:**

A 48-year-old Japanese woman with high myopia presented with decreased visual acuity. Best-corrected visual acuity was 20/28 in the right eye and 20/16 in the left eye; axial length was 29.0 mm and 28.7 mm, respectively. Fundoscopy revealed an epiretinal membrane in the left eye. Three years later, the best-corrected visual acuity in the left eye had decreased to 20/33; at this time, the patient underwent vitrectomy with epiretinal membrane and internal limiting membrane peeling in this eye. Six months later, the best-corrected visual acuity in the left eye decreased suddenly to 20/100. Optical coherence tomography showed a nodule-like lesion in the outer retina with disruption of the retinal pigment epithelium and a focally thickened choroid, compatible with PIC. One month later, the choroidal thickness had decreased. The central chorioretinal atrophy expanded rapidly at a rate of 0.45 mm^2^/year over the next 3 years, and new areas of patchy focal chorioretinal atrophy developed in the perifovea.

**Conclusions:**

Rapid progression of chorioretinal atrophy was observed in a patient with punctate inner choroiditis. Because punctate inner choroiditis is often associated with degenerative myopia, the retina is fragile and may be susceptible to mechanical damage. This case report alerts clinicians to the need for careful management of patients with punctate inner choroiditis, especially after vitrectomy.

## Background

Punctate inner choroiditis (PIC) is an inflammatory ocular disease of unknown etiology belonging to the group of idiopathic white dot syndromes. PIC is considered to be an autoimmune disease caused by factors such as infection, immunization, or stress. Most of these lesions involve the posterior pole, arising at the level of the retinal pigment epithelium (RPE) and inner choroid in the absence of anterior chamber or vitreous inflammation [1]. An unknown insult may trigger an autoimmune response against antigens in outer retina or inner choroid, leading to the development of PIC [2]. PIC is highly variable, from cases that heal spontaneously to cases that cause severe loss of vision in both eyes. It tends to present in young to middle-aged women with myopia [3, 4]. At the onset, PIC is characterized by thickening of the choroid. The typical chorioretinal inflammatory lesions occurring in PIC are seen clinically as single or multiple yellow-grayish spots in the posterior pole and peripheral retina that may progressively evolve into a punched-out atrophic scar with a variable degree of pigmentation [4, 5]. Many cases show gradual progression [4], 6, 7. There are few reports on progression rate of chorioretinal atrophy (CRA) in the long term [8, 9]. Here we report a case of rapid progression of CRA after onset of PIC.

## Case presentation

**Fig. 1:**
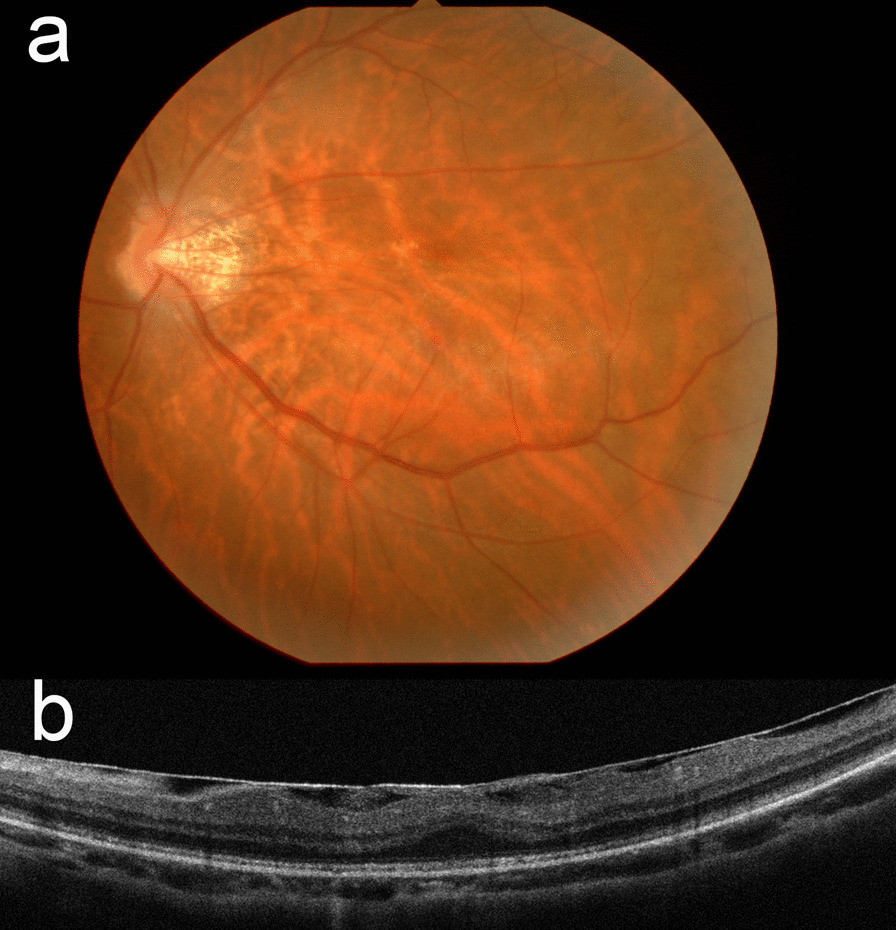
Fundus photograph and optical coherence tomography images of the left eye obtained at the first visit. **a** Epiretinal membrane and a yellow-grayish spot can be seen in the parafovea. **b** Optical coherence tomography image showing the epiretinal membrane

A 48-year-old Japanese woman with high myopia presented with decreased visual acuity. Axial length was 29.0 mm in the right and 28.7 mm in the left eyes, respectively; refractive errors were −11.5 and −10.5 diopter; best-corrected visual acuity (BCVA) was 20/28 and 20/16, respectively. The BCVA was described by converting the decimal visual acuity into fractional visual acuity. Mild cataract was observed in both eyes. Fundoscopy and optical coherence tomography (OCT) images showed epiretinal membrane (ERM) in the left eye (Fig. 1).

**Fig. 2:**
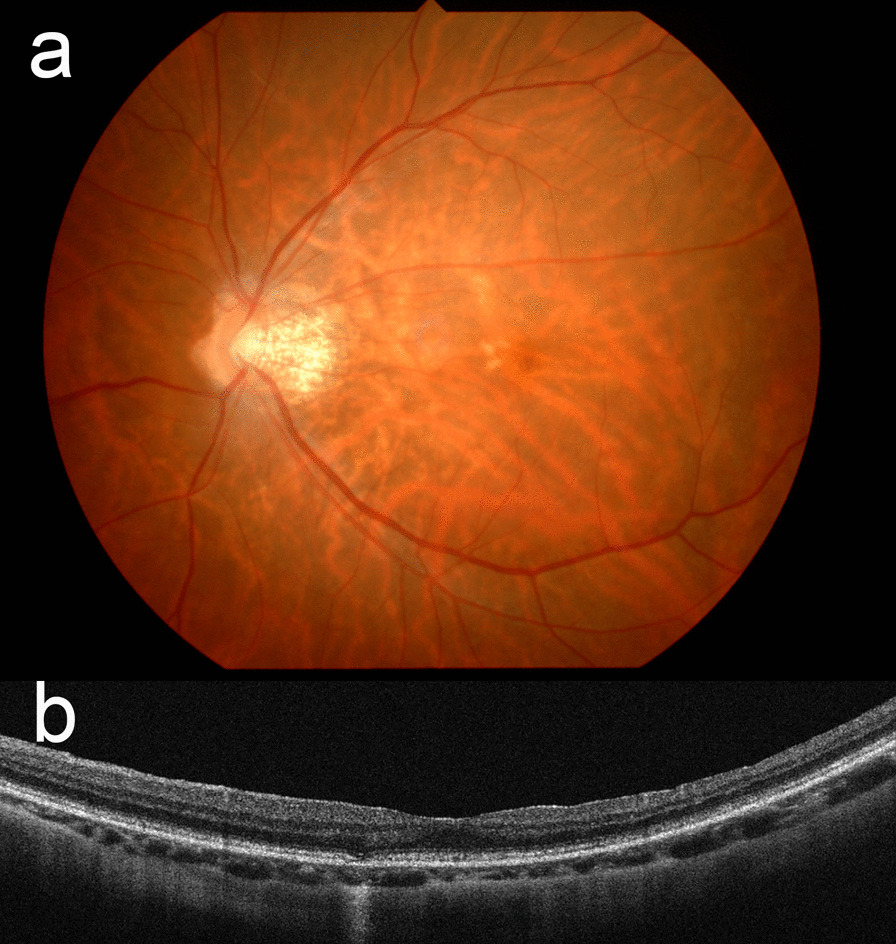
Fundus photograph and optical coherence tomography images of the left eye obtained 6 months after vitrectomy and peeling of epiretinal membrane. **a** Chorioretinal atrophy in the parafovea. **b** Optical coherence image showing irregularities of the retinal pigment epithelium at the parafovea. An increased transmission signal from the sclera can be seen under the retinal pigment epithelium, suggesting atrophy

Three years later, she developed blurred vision and BCVA in the left eye decreased to 20/33; OCT revealed thickening of the ERM. The patient opted for vitrectomy after thorough discussion and considering recent reports showing good treatment outcome for ERM with good visual acuity [10]. The patient subsequently underwent uncomplicated 25-gauge pars plana vitrectomy with ERM and internal limiting membrane (ILM) peeling in the left eye. After 6 months, BCVA was 20/28. CRA was noted in the parafovea, and OCT revealed irregularities in the RPE with increased transmission signal from the sclera, suggesting atrophy of the RPE (Fig. 2).

**Fig. 3:**
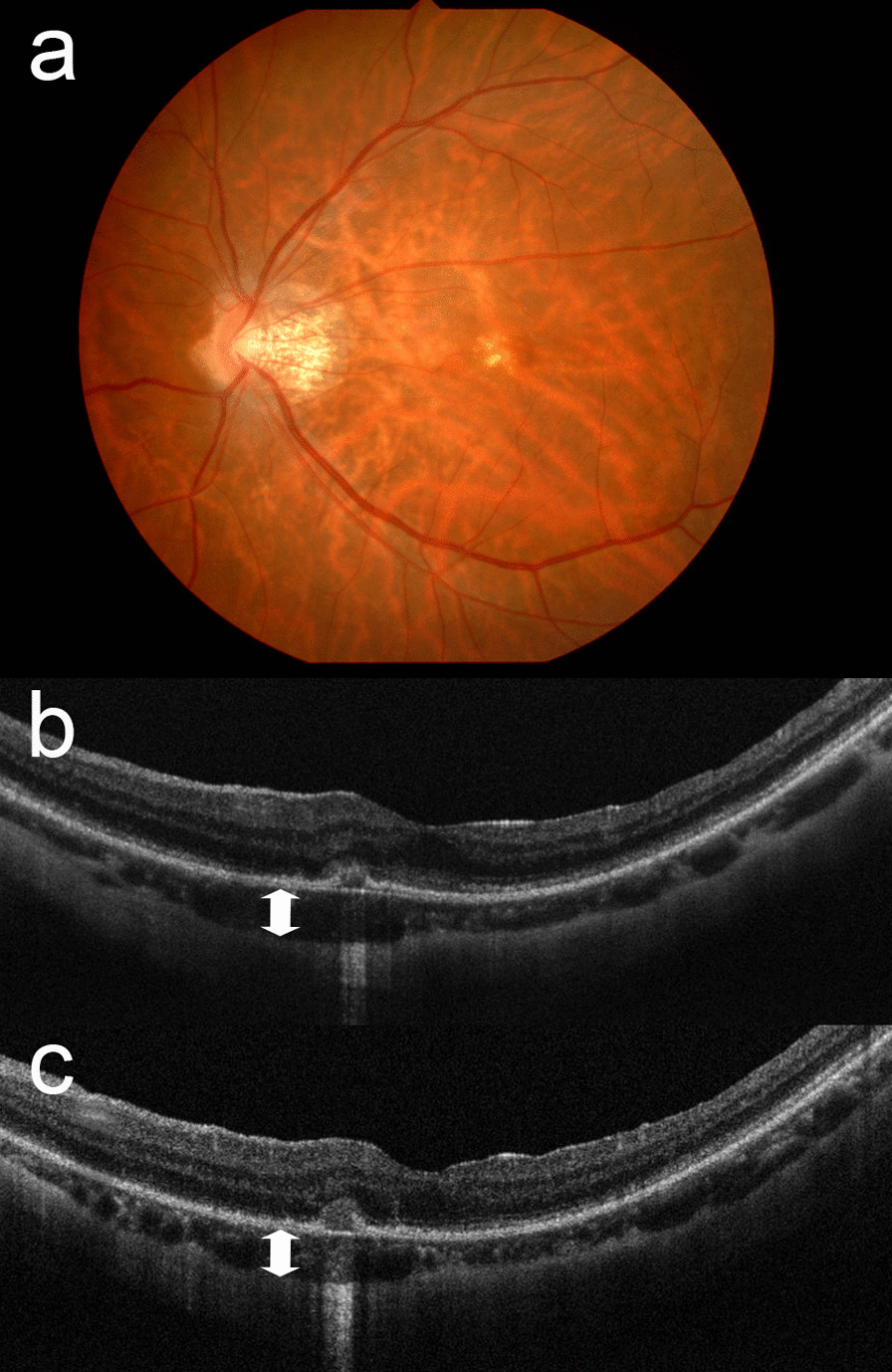
Fundus photograph and optical coherence tomography image of the left eye obtained at the time of the decrease in best-corrected visual acuity (**a**, **b**) and 2 days after an intravitreal injection of ranibizumab (**c**). **a** Chorioretinal atrophy was still present in the parafovea, but subretinal hemorrhage was unclear. **b** The retinal pigment epithelium was partially elevated and the interdigitation, and ellipsoid zones were disrupted. The choroidal thickness at this site was increased from 134 to 151 µm (between arrows). **c** Two days after injection of ranibizumab, a nodule-like lesion was noticed in the disrupted retinal pigment epithelium and the choroidal thickness had decreased to 142 µm (between arrows)

**Fig. 4:**
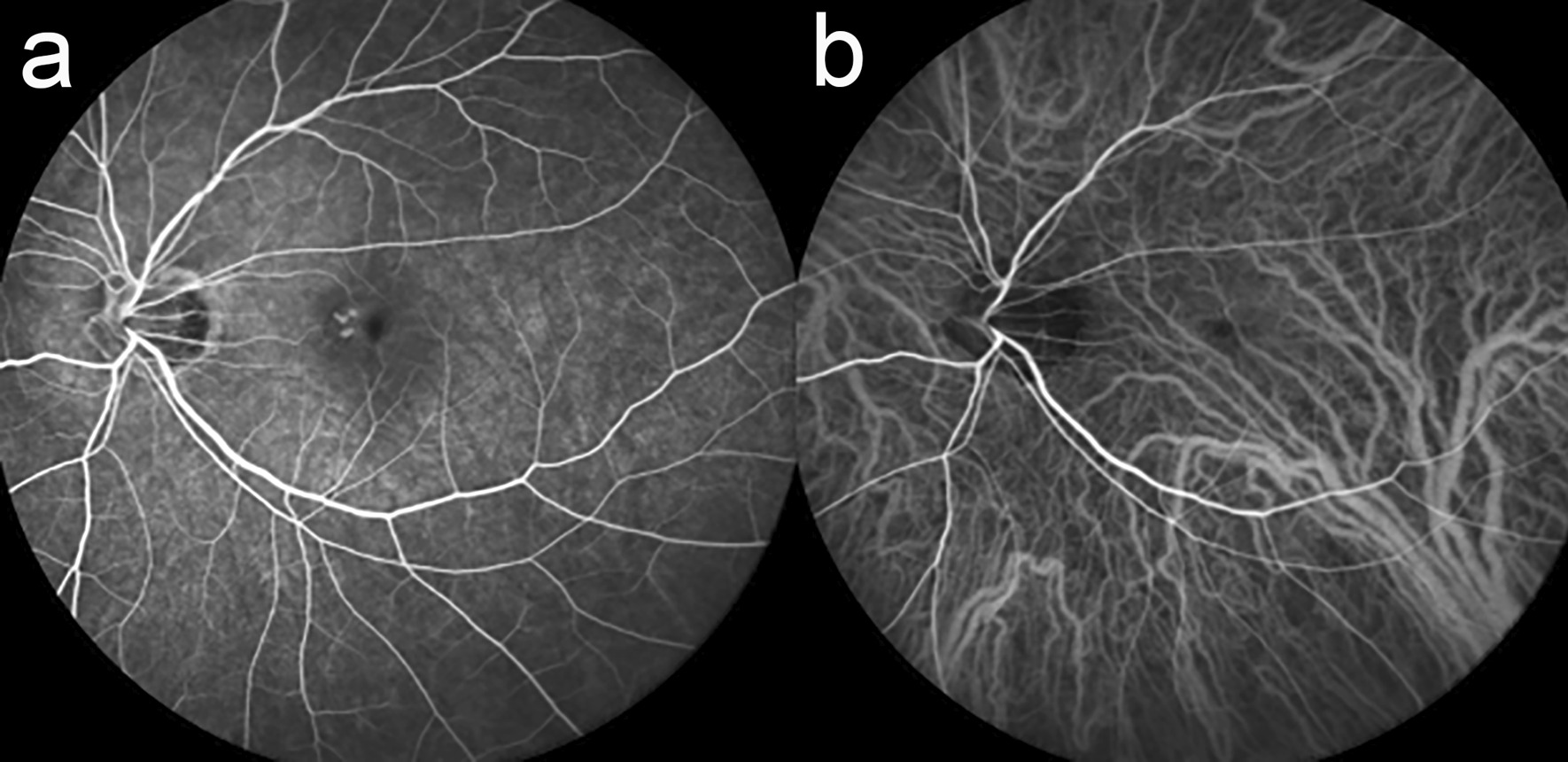
Images of fluorescein (**a**) and indocyanine green (**b**) angiography. Hyperfluorescence was observed, but no leakage. Typical choroidal neovascularization (CNV) or lacquer crack were not shown (**a**). Indocyanine green angiography did not detect abnormal blood vessels suggesting CNV (**b**)

After another 3 weeks, BCVA in the left eye decreased suddenly from 20/28 to 20/100. OCT demonstrated disruption of the interdigitation and ellipsoid zones and elevation of the RPE. Choroidal thickness at this site increased from 134 µm to 151 µm (Fig. 3a, b). Fluorescein angiography (FA) revealed hyperfluorescence (Fig. 4a), which coincided with the site where changes were observed in RPE and choroid on OCT (Fig. 3b). Hyperfluorescence was observed from an early stage, but no leak thereafter. Typical choroidal neovascularization (CNV) or lacquer crack were not shown on FA. Indocyanine green angiography (ICGA) did not detect abnormal blood vessels suggesting CNV (Fig. 4b). Although fluorescein and indocyanine green angiography did not show typical CNV or lacquer crack, myopic CNV could not be ruled out. Therefore, intravitreal ranibizumab injection was recommended. However, 2 days after the injection, the patient presented with a further decline in vision to 20/222 in her left eye. Despite no obvious change on fundus examination, OCT revealed a nodule-like outer retinal lesion with disruption of the RPE, suggesting PIC. Choroidal thickness had decreased slightly to 142 μm (Fig. 3c). Careful observation was recommended after thorough discussion with the patient concerning possible treatment options, including steroid therapy.

**Fig. 5:**
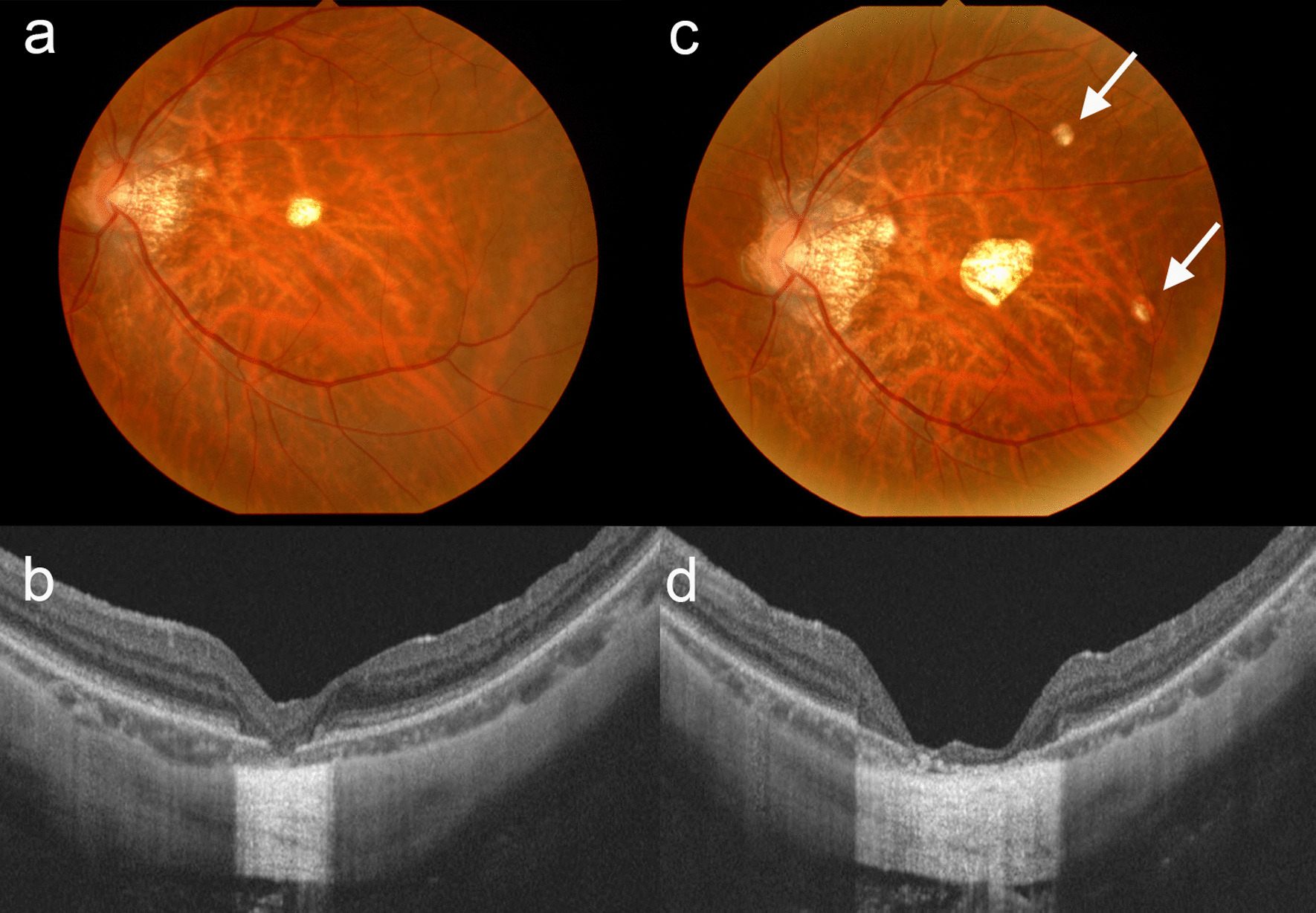
Fundus photographs and optical coherence tomography images of the left eye acquired 14 months (**a**, **b**) and 3 years (**c**, **d**) after intravitreal injection of ranibizumab. The choroidal atrophy gradually expanded, and new atrophic lesions appeared (arrows). The areas of chorioretinal atrophy measured 0.38 mm^2^ (**a**) and 1.59 mm^2^ (**c**). The choroidal thickness decreased to 52 μm (**b**) and 16 μm (**d**)

One month after the injection, BCVA improved to 20/100 and the nodule-like lesion regressed, leaving an expanded RPE defect with a decrease in choroidal thickness to 84 μm. The lesion was judged to be inactive, and further observation was recommended. However, the area of CRA increased gradually to 0.38, 0.90, and 1.59 mm^2^ and choroidal thickness decreased to 52, 40, and 16 μm by 14, 30, and 40 months post-injection, respectively (Fig. 5). CRA progressed at a rate of 0.45 mm^2^/year. The CRA was measured on color photographs of the fundus using ImageJ software (freely available at http://imagej.nih.gov/ij/). Three years after the injection, BCVA was 20/70 in the left eye and two new atrophic lesions were noted in the parafovea (Fig. 5c).

## Discussion and conclusions

PIC with a single sub-/parafoveal lesion is sometimes difficult to distinguish from myopic CNV [11], even with use of FA, ICGA, and OCT, which are the gold standard imaging modalities used to identify and monitor CNV. Recently, the usefulness of CNV detection by OCTA has been reported. Combining these four tests to detect CNV became the new golden standard. In our case, there was no OCTA in our facility at that time. Therefore, the OCTA test could not be performed. Key findings for a diagnosis of PIC include choroidal thickening [6]. When the inflammatory lesions are active, there is often mild transient focal thickening of the underlying choroid. After resolution of the acute inflammation, choroidal thinning becomes evident beneath the lesion. As proposed previously [12], choroidal inflammation damages the outer blood–retinal barrier of the RPE and triggers exudative change; in severe cases, like the one presented here, Bruch’s membrane is also damaged or ruptured. Our diagnosis of PIC in this patient was further supported by the satellite lesions that appeared years later.

To date, there is no clear consensus on the effectiveness of therapies for PIC, and there is only anecdotal evidence for the effectiveness of corticosteroids in controlling inflammation [4, 13]. Brueggeman *et al*. reported that corticosteroid therapy was associated with a decrease in the number of choroidal PIC region; however, visual acuity did not change due to subfoveal scarring [13]. In this study, diagnosis was not established initially despite choroidal thickening, and systemic steroids were not administered. There were no signs of choroidal inflammation thereafter. In hindsight, anti-inflammatory treatment could be a treatment option to retard disease progression; there is need to balance out its possible efficacy and side effects. No inflammatory findings were found in the anterior and posterior eyes other than the localized areas of the retina and choroid. Therefore, comprehensive screening for inflammatory diseases and infections of eyes was not performed. The differential diagnosis includes diseases that cause peripheral curvilinear pigmentary lesions, such as putative multifocal choroiditis, ocular histoplasmosis syndrome, and tuberculosis. However, our patient showed no evidence of previous ocular inflammation, and histoplasmosis is considered nonexistent in Japan. There was no history of tuberculosis, and chest X-ray showed no abnormalities.

Our patient developed PIC at the age of 51 years, which is an atypical age for the manifestation of PIC. PIC is usually reported as bilateral (80%) [14], but in this case it was unilateral. ERM had developed before the onset of PIC in this case. Vitreous surgery was performed because of slightly decreased visual acuity, considering recent reports showing good treatment outcome for ERM with good visual acuity [10]. ILM peeling was also performed because it is associated with a lower ERM recurrence rate [15] and there was no recurrence. Although the pathogenesis of PIC is unknown [4], the inflammatory response to surgery might have contributed to development of PIC in this case, considering the atypical presentation. Furthermore, the retina may have been mechanically stretched during ILM peeling, causing damage to the RPE or Bruch’s membrane and triggering PIC, although no obvious mechanical damage, as previously reported [16], has been observed.

In highly myopic eyes, the CRA may expand from rupture of Bruch’s membrane [17]. Long-term follow-up of patients with pathologic myopia has shown that 67.7% of patients had CRA enlargement [18]. Miera *et al*. analyzed CRA progression in patients with pathologic myopia and reported a CRA enlargement rate of 0.821 mm^2^/year [19]. This type of progression has also been reported in PIC [20]. In previous reports, the rate of progression of CRA was as rapid as 3.735 mm^2^/year [9] and 0.69 mm^2^/year [8] for the sum of multiple areas of CRA in one eye. In our case, the rate of progression was 0.45 mm^2^/year for only one area of CRA. The progression rate of CRA due to retinal photocoagulation spots in diabetic retinopathy has been reported, but the rate is 0.127 mm^2^/year, which is smaller than that of PM and PIC [9] (Table 1). We assume the progression to be rapid. In pathological myopia, the progression rate of CRA is high [19]. In our case, axial length is 28.7 mm, which is considered to be pathological myopia. This is one of the reasons why the expansion speed of CRA was fast in our case. However, the speed of expansion cannot be explained by itself. Given that the ILM had already been removed, there was no tension on the retina, which may have allowed the CRA to expand rapidly. Furthermore, the eye had no vitreous body, which would have resulted in a change in the intraocular environment, including an increase in oxygen concentration [21] and changes in intraocular cytokine levels [22], potentially accelerating the progression of atrophy.

**Table 1:** Progression rate of CRA in previous reports

Author	Disease	CRA progression rate (mm^2^/year)	Number of CRA
Miere *et al*. [19]	PM	0.821	Multiple areas
Chen *et al*. [8]	PIC	0.69	Multiple areas
Hua *et al*. [9]	PIC	3.735	Multiple areas
Hua *et al*. [9]	DR	0.127	Multiple areas
Kasuya *et al*.	PIC	0.45	Only one area

*PM* pathologic myopia, *PIC* punctate inner choroidopathy, *DR* diabetic retinopathy, *CRA* chorioretinal atrophy

PIC is a form of uveitis associated with autoimmune diseases, systemic infections, or stress. It is not a disease for which a clear association with a specific disease has been found. In our case, there was no particular autoimmune disease, systemic infection, or stress. Idiopathic ERM, which was a preexisting PIC, was also a disease peculiar to the eye and was not considered to be related to systemic diseases. However, myopia is increasing rapidly worldwide [23]. In recent years, autoimmune diseases have been included as immune-mediated inflammatory diseases (IMID), and it has become clear that inflammation is strongly related to the pathological condition of autoimmune disease. The estimated prevalence of IMID in Western society is considerable, estimated to be 5–7% [24]. It is interesting to note that severe myopic eyes that had previously undergone surgical invasion developed an inflammatory response suspected of being an autoimmune response and the atrophic lesions expanded rapidly.

We believe this case to be unique in that PIC occurred 6 months after vitrectomy with ILM peeling. To our knowledge, there are no other reports of vitrectomy and ILM peeling associated with PIC; however, we could not rule out the influence of vitrectomy and ILM peeling on the occurrence or progression of PIC. High myopia with a markedly increased axial length and vitreous surgery may be associated with rapid spread of atrophy. Given that PIC is often associated with myopia and a fragile retina, patients with PIC who undergo vitreous surgery and ILM peeling require careful follow-up.

## Data Availability

The author’s original image files are available within the article.

## Abbreviations

PIC: Punctate inner choroiditis
RPE: Retinal pigment epithelium
BCVA: Best-corrected visual acuity
CRA: Chorioretinal atrophy
OCT: Optical coherence tomography
ERM: Epiretinal membrane
ILM: Internal limiting membrane
FA: Fluorescein angiography
CNV: Choroidal neovascularization
ICGA: Indocyanine green angiography
IMID: Immune-mediated inflammatory diseases

## References

[1] Watzke RC, Packer AJ, Folk JC, Benson WE, Burgess D, Ober RR. Punctate inner choroidopathy. Am J Ophthalmol. 1984;98:572–84. 10.1016/0002-9394(84)90243-5 6208783

[2] Jampol LM, Becker KG. White spot syndromes of the retina: a hypothesis based on the common genetic hypothesis of autoimmune/inflammatory disease. Am J Ophthalmol. 2003;135:376–9. 10.1016/s0002-9394(02)02088-3 12614757

[3] Gerstenblith AT, Thorne JE, Sobrin L, Do DV, Shah SM, Foster CS, *et al*. Punctate inner choroidopathy: a survey analysis of 77 persons. Ophthalmology. 2007;114:1201–4. 10.1016/j.ophtha.2006.10.047 17434588

[4] Ahnood D, Madhusudhan S, Tsaloumas MD, Waheed NK, Leane PA, Denniston AK. Punctate inner choroidopathy: a review. Surv Ophthalmol. 2017;62:113–26. 10.1016/j.survophthal.2016.10.003 27751823

[5] Spaide RF, Goldberg N, Freund KB. Redefining multifocal choroiditis and panuveitis and punctate inner choroidopathy through multimodal imaging. Retina. 2013;33:1315–24. 10.1097/IAE.0b013e318286cc77 23584703

[6] Munk MR, Jung JJ, Biggee K, Tucker WR, Sen HN, Schmidt-Erfurth U, *et al*. Idiopathic multifocal choroiditis/punctate inner choroidopathy with acute photoreceptor loss or dysfunction out of proportion to clinically visible lesions. Retina. 2015;35:334–43. 10.1097/IAE.0000000000000370 PMC4304934 25322466

[7] Jung JJ, Mrejen S, Freund KB, Yannuzzi LA. Idiopathic multimodal imaging analysis. Retin Cases Brief Rep. 2014;8:141–4. 10.1097/ICB.0000000000000026 25372332

[8] Chen YC, Chen YL, Chen SN. Chorioretinal atrophy in punctate inner choroidopathy/multifocal choriditis: a five-year follow-up study. Occul Immunol Inflamm. 2021;8:1–6. 10.1080/09273948.2020.1869269 33684021

[9] Hua R, Liu L, Chen L. Evaluation on the progression rate of atrophy lesions in punctate inner choroidopathy (PIC) based on autofluorescence analysis. Photodiagnosis Photodyn Ther. 2014;11:565–9. 10.1016/j.pdpdt.2014.07.002 25046400

[10] Nakashizuka H, Kitagawa Y, Wakatsuki Y, Tanaka K, Furuya K, Hattori T, *et al*. Prospective study of vitrectomy for epiretinal membranes in patients with good best-corrected visual acuity. BMC Ophthalmol. 2019;19:183. 10.1186/s12886-019-1185-z PMC6693285 31412813

[11] Vance SK, Khan S, Klancnik JM, Freund KB. Characteristic spectral-domain optical coherence tomography findings of multifocal choroiditis. Retina. 2011;31:717–23. 10.1097/IAE.0b013e318203c1ef 21386760

[12] Zhang X, Zuo C, Li M, Chen H, Huang S, Wen F. Spectral-domain optical coherence tomographic findings at each stage of punctate inner choroidopathy. Ophthalmology. 2011;120:2678–83. 10.1016/j.ophtha.2013.05.012 23769333

[13] Brueggeman RM, Noffke AS, Jampol LM. Resolution of punctate inner choroidopathy lesions with oral prednisone therapy. Arch Ophthalmol. 2002;120:996. 10.1001/archopht.120.7.996 12096982

[14] Brown J Jr, Folk JC, Reddy CV, Kimura AE. Visual prognosis of multifocal choroiditis, punctate inner choroidopathy, and the diffuse subretinal fibrosis syndrome. Ophthalmology. 1996;103:1100–5. 10.1016/s0161-6420(96)30561-7 8684800

[15] Chang WC, Lin C, Lee CH, Sung TL, Tung TH, Liu JH. Vitrectomy with or without internal limiting membrane peeling for idiopathic epiretinal membrane: a meta-analysis. PLoS ONE. 2017;12:e0179105. 10.1371/journal.pone.0179105 PMC5473547 28622372

[16] Karacorlu M, Karacorlu S, Ozdemir H. Iatrogenic punctate chorioretinopathy after internal limiting membrane peeling. Am J Ophthalmol. 2003;135:178–82. 10.1016/s0002-9394(02)01925-6 12566021

[17] Ohno-Matsui JJB, Spaide RF. Macular Bruch membrane holes in highly myopic patchy chorioretinal atrophy. Am J Ophthalmol. 2016;166:22–8. 10.1016/j.ajo.2016.03.019 27018232

[18] Hayashi K, Ohno-Matsui K, Shimada N, Moriyama M, Kojima A, Hyahashi W, *et al*. Long-term pattern of progression of myopic maculopathy: a natural history study. Ophthalmology. 2010;117:1595–611. 10.1016/j.ophtha.2009.11.003 20207005

[19] Miere A, Capuano V, Serra R, Jung C, Souied E, Querques G. Evaluation of patchy atrophy secondary to high myopia be semiautomated software for fundus autofluorescence analysis. Retina. 2018;38:1301–6. 10.1097/IAE.0000000000001733 28574923

[20] Zhang X, Wen F, Zuo C, Li M, Chen H, Huang S, *et al*. Clinical features of punctate inner choroidopathy in Chinese patients. Retina. 2011;31:1680–91. 10.1097/IAE.0b013e31820a67ad 21499193

[21] Holekamp NM, Shui YB, Beebe DC. Vitrectomy surgery increases oxygen exposure to the lens: a possible mechanism for nuclear cataract formation. Am J Ophthalmol. 2005;139:302–10. 10.1016/j.ajo.2004.09.046 15733992

[22] Gu R, Zhou M, Jiang C, Yu J, Xu G. Elevated concentration of cytokines in aqueous in post-vitrectomy eyes. Clin Exp Ophthalmol. 2016;44:128–34. 10.1111/ceo.12638 26317489

[23] Holden BA, Fricke TR, Wilson DA, Jong M, Naidoo KS, Sankaridurg P, *et al*. Global prevalence of myopia and high myopia and temporal trends from 2000 through 2050. Ophthalmology. 2016;123:1036–42. 10.1016/j.ophtha.2016.01.006 26875007

[24] El-Gabalawy H, Guenther LC, Bernstein CN. Epidemiology of immune-mediated inflammatory diseases: incidence, prevalence, natural history, and comorbidities. J Rheumatol Suppl. 2010;85:2–10. 10.3899/jrheum.091461 20436161

